# Design of a temperature-feedback controlled automated magnetic hyperthermia therapy device

**DOI:** 10.3389/fther.2023.1131262

**Published:** 2023-02-27

**Authors:** Anirudh Sharma, Avesh Avinash Jangam, Julian Low Yung Shen, Aiman Ahmad, Nageshwar Arepally, Hayden Carlton, Robert Ivkov, Anilchandra Attaluri

**Affiliations:** 1 Department of Radiation Oncology and Molecular Radiation Sciences, The Johns Hopkins University School of Medicine, Baltimore, MD, United States,; 2 Department of Mechanical Engineering, School of Science, Engineering, and Technology, The Pennsylvania State University—Harrisburg, Middletown, PA, United States,; 3 Department of Mechanical Engineering, Whiting School of Engineering, Johns Hopkins University, Baltimore, MD, United States,; 4 Department of Materials Science and Engineering, Whiting School of Engineering, Johns Hopkins University, Baltimore, MD, United States

**Keywords:** magnetic hyperthermia therapy, temperature control, proportional-integral-derivative (PID) control, verification, finite element analysis, safety, FDA-food and drug administration

## Abstract

**Introduction:**

Magnetic hyperthermia therapy (MHT) is a minimally invasive adjuvant therapy capable of damaging tumors using magnetic nanoparticles exposed radiofrequency alternating magnetic fields. One of the challenges of MHT is thermal dose control and excessive heating in superficial tissues from off target eddy current heating.

**Methods:**

We report the development of a control system to maintain target temperature during MHT with an automatic safety shutoff feature in adherence to FDA Design Control Guidance. A proportional-integral-derivative (PID) control algorithm was designed and implemented in NI LabVIEW^®^. A standard reference material copper wire was used as the heat source to verify the controller performance in gel phantom experiments. Coupled electromagnetic thermal finite element analysis simulations were used to identify the initial controller gains.

**Results:**

Results showed that the PID controller successfully achieved the target temperature control despite significant perturbations.

**Discussion and Conclusion:**

Feasibility of PID control algorithm to improve efficacy and safety of MHT was demonstrated.

## 1 Introduction

Magnetic hyperthermia therapy (MHT) is a potent cancer therapy that employs heat generated by magnetic nanoparticles (MNPs) embedded within the target tissue when they are exposed to an alternating magnetic field (AMF), at low radiofrequency (RF), typically <300 kHz. In 2010, the European Medicines Agency approved MHT to treat recurrent glioblastoma (GBM) in combination with fractionated radiation therapy (RT). MHT is also being explored as an adjuvant therapy for other solid tumors such as prostate (Johannsen et al., 2007, 2009; Attaluri et al., 2015), pancreas (Maluta et al., 2011; Attaluri et al., 2021; Beola et al., 2021), bone (Matsumine et al., 2011) and liver (Moroz et al., 2002; Sun et al., 2013; Attaluri et al., 2016). To date no clinical trial has been conducted in the United States with automated feedback temperature or thermal dose control for MHT.

MHT requires delivery of MNPs to the tumor and application of AMF to generate local hyperthermia (41°C–46°C) *via* magnetic hysteresis loss. Heat generated by MNPs can be quantitatively estimated through calorimetric methods and is often expressed as specific loss power (SLP) (Bordelon et al., 2011; Andreu and Natividad, 2013; Dennis and Ivkov, 2013; Dennis et al., 2015; Soetaert et al., 2017). For many MNPs, the SLP exhibits a response that is linear with AMF frequency and non-linear with AMF amplitude (Dennis and Ivkov, 2013; Dennis et al., 2015). This implies that by controlling AMF amplitude at fixed frequency, power deposition and hence temperature within the tumor near the MNPs, can be controlled.

On the other hand, MHT delivery can be challenging because MNP distribution within the tumor is typically variable and heterogeneous, leading to unpredictable temperature variations within the tumor and at tumor margins (Attaluri et al., 2011; Attaluri et al., 2011; LeBrun et al., 2013; Kandala et al., 2018). Heterogeneous MNP distribution within the tumor and the tumor microenvironment (TME) arise from aberrant tumor physiology and physical properties. New approaches are needed to achieve target hyperthermic temperatures within the TME while minimizing locally under-treated and ablative tumor zones, under-treated margins, and inadvertent heating of healthy tissue (Moroz et al., 2002; Kandala et al., 2018; Darvishi et al., 2021; Singh et al., 2021; Tansi et al., 2021).

Computational methods predict improved spatiotemporal control of treatment temperature with AMF amplitude and power modulation (Soetaert et al., 2015, 2020; Kandala et al., 2018; Orrico et al., 2022). Success with these approaches requires real-time temperature monitoring to provide the needed input into a temperature controller device. Among the temperature control methods tested, proportional-integral (PI) or proportional-integral-derivative (PID) control has demonstrated utility in preclinical tests for laser ablation and focused ultrasound hyperthermia (Loulou and Scott, 2002; Hartov et al., 2009; Korganbayev et al., 2020, 2021; Bianchi et al., 2021; Ahmed et al., 2022; Tenorio et al., 2022). However, automated temperature control in clinical hyperthermia systems, particularly for MHT, remains underdeveloped. It is expected that the FDA will classify a feedback temperature controller device for MHT as a significant risk device because it has direct control over energy delivery that has potential to damage tissue. Thus, adherence to FDA’s Design Control Guidance during device design and development is critical to gain regulatory approval for a clinical trial (www.fda.gov) (FDA.gov, 2022). This process facilitates medical device implementation with careful risk management strategies to enable thorough evaluation ensuring safe and reliable performance.

Herein, we document the design process for an experimental PID-controlled automated MHT device that uses fiber-optic temperature data as input to manage power delivered to a custom-designed 20 cm diameter RF coil connected to a 120 kW induction heating power supply. The intended use is treating canine glioblastoma in a future pilot study. We describe here the details of the controller design, its development, and verification of its performance to specified criteria. Device design inputs comprised: (i) AMF power management to achieve user-specified set point temperature quickly; (ii) risk management with user-defined safety temperature thresholds; and, (iii) reliability inputs to manage temperature and controller stability, reproducibility, and robustness. These requirements translated into the following controller device design parameters: (i) rise time (*t*_*r*_) < 1 min and settling time (*t*_*ss*_) < 5 min; (ii) initial overshoot (*M*_*p*_) < 5%, an overriding 0 V signal when safety thresholds are exceeded; and, (iii) choice of PID control for stability and robustness. Temperature thresholds for safety were defined in the controller operation to minimize off-target heating and to prevent tissue ablation. Design input and output definitions, design verification and review conformed to FDA Device Control Guidance for Medical Device Manufacturers (www.fda.gov) (FDA.gov, 2022). Results of trials validating controller performance to its intended end use in a living subject were published elsewhere (Sharma, et al., 2023).

## 2 Methods

The device was developed in adherence to the US Food and Drug Administration (FDA) Design Control Guidance for Medical Device Manufacturers, Document 21 CFR 820.30 (FDA.gov, 2022). Our approach incorporated the design-review-verification-review-validation approach of design control to assure the device design performs to its intended use. Project team members were divided into two groups, representing a development team and a verification/validation team to provide critical feedback to verify that design outputs met design inputs. Regular formal project meetings were conducted to review design, control design changes, review design results, and discuss potential changes to the device design based on feedback from verification or failure modes and effects analysis (FMEA). Minutes from both device design review meetings and device design change meetings were documented and are incorporated into device design history files for future review by regulatory bodies in anticipation of human clinical product development to ensure device design is correctly translated into production specifications.

### 2.1 Controller design workflow and design requirements

**FIGURE 1:**
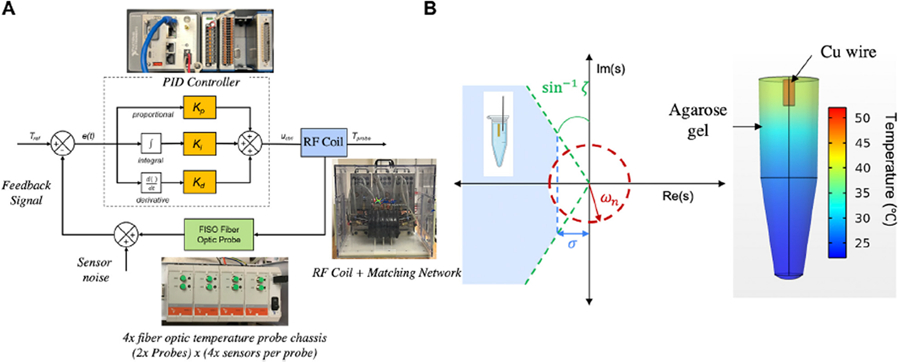
**(A)** Schematic block diagram of the PID controller depicting the error signal (*e(t)*) input and control signal (*u*_*ctrl*_) generation to minimize the error *e(t)* using the PID algorithm. Adapted Jangam et al. (2022), with permission from SB3C Foundation, Inc. Reproduced under CC-BY-4.0, Sharma et al (2023). **(B)** Regions in the *s*-plane, delineated by transient specifications on controller design, including rise time (*ω*_*n*_, red), overshoot (*ζ*, green) and settling time (*σ*, blue). The agarose gel + Cu wire system (inset schematic) must constitute a transfer function with poles in the shaded region to meet design specifications. The 3D temperature color graph indicates the temperature distribution in the sample after 10 min of AMF treatment at 123 Oe (peak), 160 kHz. Reproduced under CC-BY-4.0, Sharma et al (2023).

The controller design workflow involved defining performance and safety requirements as design inputs, device hardware and software development, verification of controller responses *in vitro* systems with failure analysis, 3D computational modeling to estimate PID gains, and experimental validation of the feedback control based on computed PID gains (Figure 1A and Supplementary Figure S1).

The design requirements for a temperature feedback controller for MHT include satisfying general hardware and software compatibility, safety requirements, and MHT treatment-specific performance requirements. For the present case, these were:

1. MHT treatment and controller performance criteria:
  - Capability to maintain temperature at a target set point temperature (*T*_*ref*_) for 15–30 min at a single probe location.
  - Capability to achieve hyperthermic temperatures (43°C–45°C), and maintain temperature at setpoint (*T*_*ref*_) to attain thermal dose defined by the metric cumulative equivalent minutes at 43°C (CEM43) of 60 ± 5 min *within clinically relevant treatment times* (15–30 min).
  - Rise time (*t*_*r*_) to target temperature (*T*_*ref*_) < 60 s.
  - Overshoot (*M*_*p*_) = < 5%.
  - Settling time (*t*_*ss*_) within ±0.5°C in <5 min.
2. Safety criteria:
  - A defined maximum temperature (e.g., 50°C) within the treatment region to limit power to prevent runaway heating at the feedback sensor location.
  - A safety temperature threshold at a distant location for additional safety monitoring, for example a temperature representing body core temperature.
3. Hardware and software requirements:
  - Integration with multi-sensor temperature probe to provide temperature readout capability with appropriate sampling interval.
  - Prevent power fluctuations that might arise from higher-order harmonics generated by the RF power supply that can damage electronics in the 120 kW AMF.

### 2.2 Controller performance criteria

In general, for effective hypothermia treatment (HT), therapeutic heating must be maintained for the duration of treatment, or at least 15–30 min to achieve clinically relevant thermal doses, i.e., CEM43 about 30 to 60 min. An isoeffect thermal dose target of CEM43 greater than 30 min in 90% of the tumor volume i.e., CEM43T90 with HT alone correlates with favorable treatment outcomes for many tumors independent of HT modality, although HT is rarely administered as a single agent therapy (Franckena, M. et al., 2009; Dewey, 2009; Dewhirst, et al., 2003; Maluta, et al., 2011; Rhoon, 2016). In this study, Requirements 1(c)-1(e) were chosen to maximize time at therapeutic temperature CEM43 (Dewey, 2009; Rhoon, 2016). These transient response requirements translated into specifications in the *s*-domain (Laplace domain) to define the parameter space over which the controller should be stable. The closed-response response for the temperature control process (eventually for MHT) is thus defined by a second-order transfer function (Ebert et al., 2010; Kandala et al., 2018),

(1) $$Q\left(s\right)=\;\frac{{\omega_{n}}^{2}}{\left(s^{2}+2\zeta \omega_{n}+\;\omega_{n}^{2}\right)}$$

which has the poles$-\sigma \pm j\omega_{d}$, and

(2) $$\sigma =\;\zeta \omega_{n}$$

(3) $$\omega_{d}=\;\omega_{n}\sqrt{\left(1-\zeta^{2}\right)}$$

ζ, a unitless damping ratio (0 ≤ ζ ≤ 1), *ω*_*n*_ is the (undamped) natural frequency in rad·s^−1^, and ***σ*** has units s^−1^. The following constraints were imposed as design specifications:

(4) $$t_{r}<60\;s,t_{r}=\;\frac{1.8}{\omega_{n}}\to \;\omega_{n}>0.03\;rad\cdot s^{-1}$$

(5) $$t_{ss}<5\;min,t_{ss}\;=\frac{4.6}{\sigma }\to \;\sigma >0.015\;s^{-1}$$

(6) $$M_{p}<5\%,\;M_{p}=\;e^{\frac{-\pi \zeta }{\sqrt{\left(1-\zeta^{2}\right)}}}\to 0.7<\zeta <1.0$$

Values representing the poles of the closed-loop transfer function must satisfy these requirements, and therefore must be located in the left-hand side of the *s*-plane (LHP) as shown in Figure 1B. Full derivation of the second order transfer function is defined by the experimental system, as constrained by the above requirements, and is described in Section 2.5.

### 2.3 Safety controls requirements

To meet requirements 2(a)-(b), a LabVIEW^®^ code (National Instruments, Austin, TX) was developed to include a user-defined safety threshold temperature (Supplementary Figure S1), *T*_*threshold*_ (°C). If exceeded by the measured temperature at the sensor, *T*,a0 V analog signal is sent to the power supply to discontinue heating by reducing current delivered to the AMF coil to 0 A within 0.2 s, until the temperature at the probe satisfied *T* < *T*_*threshold*_. To meet safety requirements at a distant site, additional user-input temperature thresholds were accommodated in the LabVIEW program. The safety temperature probe(s) were connected to a separate FISO TMI4 temperature conditioner (FISO, Quebec, CA) to record the temperature, independent of the controller, as a risk management requirement (in case of feedback sensor damage). Any of these safety probes could be placed by the user (eg. subcutaneous near the skull or rectal temperature) to monitor tissue surface or physiological temperatures, respectively, during a MHT treatment. Finally, the power supply has a manual override emergency stop push-button to immediately discontinue power in case of controller failure or an emergency.

### 2.4 Controller hardware

For temperature readout (from the FISO EVO^®^ analog output) and control of the 120 kW AMF, a NI CompactRIO (NI cRIO 9042) (National Instruments, Austin, TX) with the multifunction I/O module (NI9381, with 8 analog inputs and 8 analog outputs) and compatible LabVIEW software interface (National Instruments, Austin, TX) were used. The precision and accuracy of the digitized temperatures measured by the controller were benchmarked against the FISO EVO^®^ SPC-HR reading modules (FISO, Quebec, CA), which were previously calibrated and certified by FISO for meeting QC/QA standards, i.e., S/N ≥ 69 dB. For similar precision and offset <0.3 °C vs. FISO for accuracy, the controller was shielded against RF interference (RFI) and from high frequency noise and DC offsets induced by external electromagnetic interference (EMI) from the RF coil and other external EMI sources. The controller was shielded by enclosing it within a Cu/Ni conductive mesh (Amazon, Seattle, WA), and the entire controller + shield were enclosed in a metallic cabinet (Global Industrial, Port Washington, NY) to shield the system from RFI. Temperature measurements were performed by all four sensors (T1-T4), spaced 2 cm apart)of the FISO EVO probe, in a cylindrical agarose gel sample to measure the increase in temperature from Joule heating at different radial distances from the gel center. All connecting wires were shielded and shielding was electrically grounded to prevent offsets from ground loops.

### 2.5 Controller software

**FIGURE 2:**
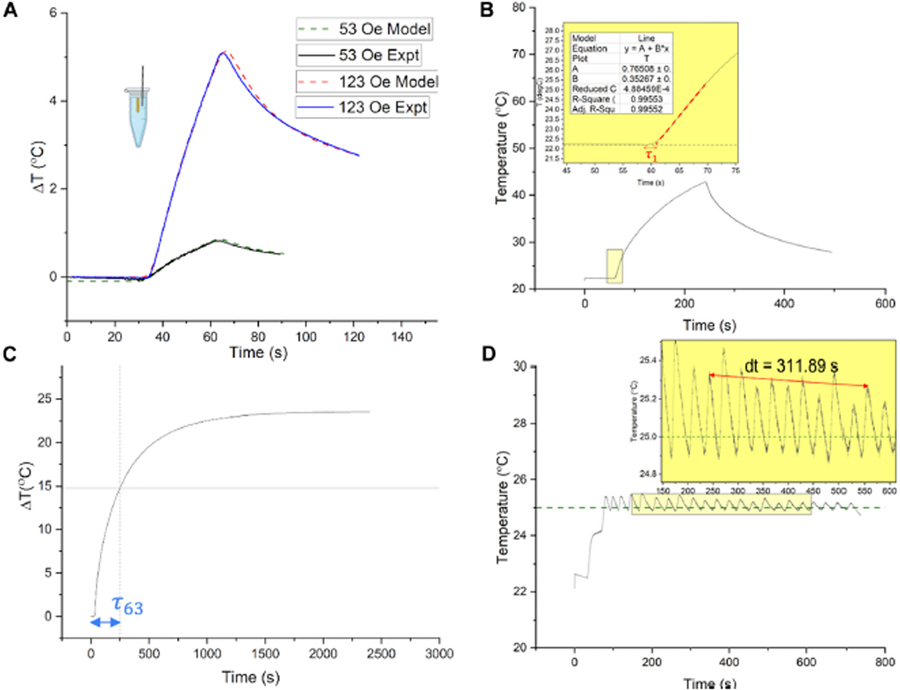
**(A)** Experimental validation of simulated open loop temperature change vs. time responses in the agarose gel + Cu wire system, to a 30 s AMF pulse. The responses were tested to 30 s AMF pulses of amplitude 4.2 kA/m and 9.8 kA/m peak field (160 kHz), respectively, which define the limits of the AMF field range used in PID control (4.2–9.8 kA/m peak, 160 kHz). Solid lines indicate experimentally measured temperature vs. time curves during heating (AMF ON) and cooling (AMF OFF) at 53 Oe (4.2 kA/m, lower curve) and 123 Oe (9.8 kA/m, upper curve). Dashed lines are obtained from the finite element analysis (FEA) agarose gel + Cu wire model, with the lower dashed curve corresponding to AMF amplitude of 53 Oe (4.2 kA/m) and upper dashed curve corresponding to AMF amplitude of 123 Oe (9.8 kA/m). **(B,C)** Estimation of heating time constants, from open loop temperature vs. time responses of the agarose gel + Cu wire system, for evaluation of PID gains for feedback control. **(D)** Closed loop temperature vs. time response of gel + Cu wire system with proportional gain, used to evaluate the undamped natural frequency of the system, *ω*_*n*_ ~ 0.2 *rad*/*s*.

LabVIEW^®^ controller code was developed to convert measured analog temperatures to digital signals, *T(t),* that were then used to compute error, *e(t),* as the difference between user-defined set-point temperature, *T*_*ref*_, and *T(t)*, and then to calculate the new control signal, *u*_*ctrl*_*(t),* using the digital LabVIEW^®^ PID algorithm and user inputs of proportional, integral and derivative gains *K*_*p*_*, K*_*i*_*, K*_*d*_, respectively. The frequency of these operations was determined by the sampling interval used to digitize the temperature signal, FPGA clock rate (40 MHz), and frequency with which the power supply could respond to (0–5 V) analog signals without generating a fault. Digital signal processing included a four-pole low-pass Butterworth filter with a cut-off frequency of 100 Hz (<< 0.5*10,000 samples per second sampling rate, to prevent aliasing), which improved the signal-to-noise (S/N) ratio to 57.6 dB and resulted in a Gaussian distribution of noise. The fastest dynamics of the plant are expected to be at least an order of magnitude lower than the cutoff frequency, so no information would be lost to filtering. Next, a DC averaging filter was used to average 1,000 samples collected in every 100 ms interval to reduce the high frequency Gaussian noise and improve the S/N comparable to the FISO-SPC-HR modules (~69 dB). The processed signal was updated every 100 ms. The interval of 100 ms was decided based on the (assumed) fastest dynamics of the system and the rated response time of the temperature sensor (<100 ms). For example, assuming the fastest dynamics (*t*_*r*_) of 0.5 s, the calculated bandwidth is approximated by 0.35/*t*_*r*_ = 0.7 Hz, or a period of 1.42 s. Generally, it is advisable to have a sampling of the signal at a frequency of 10 times or higher than the fastest dynamics in the system (Koivo et al., 1983). Thus, we get 1.42/10 = 142 ms as a recommended sampling time. Therefore, 100 ms was used as the interval for collecting temperature data for the digitized temperature signal. The cRIO 9042 (1.6 GHz quad-core) embedded controller had the computational capacity to match this sampling rate and generate the control signal. The control signal was then converted back to an analog signal (0–5 V) and sent by the cRIO controller to the analog input terminal of the 120 kW power supply, to control voltage applied to the RF coil (which scales linearly with AMF voltage, Figure 2A inset). Temperature, power and CEM43 data were exported from the cRIO controller in TDMS format. Additionally, the upper and lower power supply bounds (0–5 V range corresponding to 0%–100% power), which are used to define the AMF amplitude range, are specified in the program by the user based on performance and safety considerations (eddy current heating, thermal runaway, electrical safety, and prevent power fault). 0.25–1.25 V (4.2–9.8 kA/m peak at 160 kHz) controller analog output range complied with these safety requirements while meeting heating performance requirements for experiments conducted in this study.

### 2.6 Modeling PID-based feedback temperature control and PID gain estimation

#### 2.6.1 Experimental electromagnetic and heat transfer model

An agarose gel + Cu wire heat source model was prepared for experimental validation by comparison with computational predictions. Briefly, a 1 ml gel model made of 1% agarose powder (Sigma-Aldrich, Burlington, MA) dissolved in 1x phosphate buffered saline (PBS) solution (Corning, Manassas, VA) was prepared in an Eppendorf tube containing a NIST traceable Cu standard wire (ESPI metals) weighing 0.104 g embedded in the center as shown in Figure 1B. PBS based agarose gels have been previously used as tissue mimicking phantoms for various electromagnetic applications. Their physical, electrical and thermal properties are well defined (Baker-Jarvis, J. et al., 2010), and they provide a medium to support the Cu wire heat source.

A Cu wire with known purity, weight and dimensions serves as an excellent standard model for induction heating, as the power deposited for a given AMF having defined frequency and amplitude can be calculated analytically and computationally (Attaluri et al., 2013). Therefore, heat transfer models employing Cu wire as heat sources, exposed to AMF, can be experimentally validated.

The sample holder was a 3D printed polylactic acid (PLA) based platform (Supplementary Figure S2A) that only contacts the Eppendorf tube containing the gel sample at the rim of the tube. The gel sample was otherwise suspended in air with no contact to the sample holder (Supplementary Figures S2B, C). Therefore, heat conduction losses to the sample holder were assumed negligible with the surrounding air assumed to act as an insulator. The temperature probe was placed 1.3 mm from the Cu wire surface.

#### 2.6.2 Computational electromagnetic and heat transfer model

The agarose gel + Cu wire system was modeled using commercial finite element analysis (FEA) software COMSOL Multiphysics (COMSOL Inc., Burlington, MA) to characterize the open loop temperature vs. time response to a step increase in magnetic field amplitude. Coupled electromagnetic and heat transfer simulations with PID temperature control at the probe location were performed on a model of the gel + Cu wire system using FEA in the following way. The geometry of the Eppendorf tube containing 1% agarose was modeled in a commercial computer aided design software and imported into the FEA software. A cylindrical uniform AMF region, equivalent in dimensions to the 20 cm diameter coil, was designed to enclose the model agarose gel + Cu wire at the center of the model coil. Power deposited in the Cu wire are governed by Maxwell’s electromagnetic wave equations, where the magnetic vector potential, *A*, is solved in each time step and the electric field intensity and current density are calculated using the following equations:

(7) $$j\omega \sigma A\;+\;\nabla \;\times \;\left(\mu^{-1}\nabla \;\times \;A\right)=\;0\;\;\;\;\;\;E=\;-j\omega A\;\;\;\;\;\;J=\;\sigma E+j\omega D$$

subject to the initial condition A = 0. *J* is the current density in A/m, *ω* is the angular frequency in rad·s^−1^, *σ* is the electrical conductivity in S/m, and *A* is the magnetic vector potential in Wb/m. Heat transfer in the sample is governed by the following equations:

(8) $$\rho C_{p}\frac{\partial T}{\partial t}=k\nabla^{2}T+Q_{SAR}$$

where *T* is temperature in K, *ρ* is the gel density in kg·m^−3^, *C*_*p*_ is the specific heat in J·kg^−1^·K^−1^, *k* is the thermal conductivity in W·m^−1^·K^−1^ and *Q*_*SAR*_ is the heat generated in the Cu wire from inductive heating in W·m^−3^·T The time average over one cycle is given by

(9) $$Q_{SAR}=\frac{1}{2}\sigma {\left|E\right|}^{2}$$

Where *E* is the electric field intensity (V·m^−1^).

The model gel is then subject to the convective cooling boundary condition,

(10) $$q=h_{conv}\cdot \left(T-T_{\infty }\right)$$

where *h*_*conv*_ is the convective heat transfer coefficient in W·m^−2^·K^−1^, and *T*_∞_ is the ambient temperature in K (Table 1 and Supplementary Figure S2D). The convective heat transfer coefficient was estimated from fitting modeled responses to experimental temperature vs. time heating and cooling responses to AMF (off)_pulses (Figure 2A).

#### 2.6.3 Sensitivity analysis and uncertainty propagation in the computational model

Sensitivity analysis for the mesh size and time-step discretization was carried out for all models to ensure accurate solutions. For the transient solution, changing the time step from 1 s to 0.5 s had negligible (<1% change) effect on the probe temperature. Increasing the mesh size from 181,133 tetrahedral domain elements to 946, 943 mesh elements resulted in <5% change in the probe temperature but increased the computational time significantly. Therefore, 181,133 tetrahedral domain mesh elements with minimum element size of 4.5 × 10^−4^ m were used.

A sensitivity analysis and uncertainty quantification of probe temperature vs. time as a function of various input parameters (applied magnetic field amplitude (*H*_*gel*_), probe distance from the Cu wire (*dist*), thermal conductivity (*k*_*gel*_), electrical conductivity of gel (*σ*), specific heat (*C*_*p*_), and density (*ρ*_*gel*_) was conducted using the uncertainty analysis module in the FEA software. The Morris one-at-a-time (MOAT) (Balesdent et al., 2016) method for qualitative sensitivity screening was used. This sensitivity analysis assigns relative weights, MOAT mean and MOAT standard deviation, to each input parameter based on the variation of the variable of interest, temperature at probe location, to variation in the input parameter, while keeping all other parameters fixed at baseline. A scatter plot of the input parameters, with the MOAT mean as the *x*-axis and the MOAT standard deviation as *y*-axis, is generated to compare relative sensitivities.

Sobol and correlation analysis for uncertainty quantification were performed. Uncertainty propagation was evaluated using Monte Carlo simulations on the surrogate model generated by FEA software. The Sobol method quantitates the fractional contribution of each input parameter (and its distribution, e.g., *H*_*gel*_, *dist*) to the variance of the probe temperature.

#### 2.6.4 PID gains estimation for closed loop temperature control

The plant, i.e., the gel + Cu wire system, is defined as a second order transfer function,

(11) $$P\left(s\right)=\;\frac{g}{\left(\tau_{1}s+1\right)\left(\tau_{2}s+1\right)}$$

where *g* is the static gain for step input in K·V^−1^, and *τ*_1_ and *τ*_2_ are time constants in s.

The parameters (*g*, *τ*_1_ and *τ*_2_) for the plant transfer function, *P*, were assessed from the open loop pulse and step responses. The static gain (*g*), and slow and fast time constants (*τ*_1_ and *τ*_2_), were evaluated from this open-loop step response, to define the plant open-loop transfer function (Eq. 11). The temperature probe was placed at 1.3 mm from the surface of the Cu wire (2.3 mm from tube center). The static gain, *g*, is given by the ratio of temperature gain achieved with the step increment in control input,$g=\frac{\Delta T}{u_{ctrl}}$. *g* was calculated from the temperature vs. time response at the probe location to a step increase in magnetic field amplitude to 123 Oe (peak) at 160 kHz (24% step increase in power) in the model. The controller PID gain constants are then derived through the following equations (Kandala et al., 2018):

(12) $$C\left(s\right)=\frac{Q\left(s\right)}{\left(1-P\left(s\right)Q\left(s\right)\right)}$$

But for PID control,

(13) $$C\left(s\right)=K_{p}+\;\frac{K_{i}}{s}+\frac{k_{d}s}{\left(1+\tau_{d}s\right)}$$

Thus,

(14) $$K_{p}=\;\kappa \left(\tau_{1}+\;\tau_{2}\;-\;\tau_{d}\right)\left(K^{-1}\right)$$

(15) $$K_{i}=\kappa \left(s^{-1}K^{-1}\right)$$

(16) $$K_{d}=\kappa \left(\tau_{1}-\;\tau_{d}\right)\left(\tau_{2}-\tau_{d}\right)\left(s\cdot K^{-1}\right)$$

where

(17) $$\kappa =\;\frac{\omega_{n}}{2\zeta g}\left(s^{-1}\cdot K^{-1}\right)$$

(18) $$\tau_{d}=\frac{1}{2\zeta \omega_{n}}\left(s\right)$$

PID gains were calculated for an ideal response assuming a critically damped system with *ζ* = 1 and *ω*_*n*_ = 0.2 *rad*/*s* (t_r_ = 7.8 s), both of which satisfy the general criteria set in Eqs 1–3 The value of |*ω*_*n*_| agrees with the undamped frequency of the system (average 31.1 s period, Figure 2D). The calculated parameters and PID gains are shown in Table 2. Full validation of PID gains with biological models is described in detail elsewhere (Sharma et al., 2023).

#### 2.6.5 Simulation of closed loop responses

To simulate the closed-loop performance of the controller in attaining the set point, *T*_*ref*_, for the calculated PID gains, the temperature vs. time responses are simulated for the agarose gel + Cu wire system in FEA software, by designating the probe temperature as the control variable and defining the set point, *T*_*ref.*_, as 25°C.

The PID controller can be described by the following equations

(19) $$u_{ctrl}\left(t\right)=K_{p}\cdot e\left(x,y,z,t\right)+K_{i}\cdot \int_{0}^{t}e\left(x,y,z,t\right)\cdot dt+K_{d}\frac{\partial e\left(x,y,z,t\right)}{\partial t}$$

where *e* (*x*,*y*,*z*,*t*) is the error signal, generated from the difference between the measured temperature at the probe location at time *t*, *T(x,y,z,t)*, and the set point temperature *T*_*ref.*_. *u*_*ctrl*_(*t*) is the normalized control signal generated by PID controller. In the model, the control signal is applied to the alternating magnetic field (AMF) amplitude such that

(20) $$H\left(t\right)=u_{ctrl}\left(t\right)\cdot H_{max}$$

where *H*_*max*_ is the upper limit of the AMF (123 Oe peak, 160 kHz). Thus, the time dependent amplitude modulation of the AMF is regulated by the control signal. The AMF amplitude is defined as spatially uniform within the coil geometry. Temperature vs. time responses are simulated for the following *(K*_*p*_*, K*_*i*_*, K*_*d*_) combinations in the neighborhood of calculated PID gains: (0.26, 1 × 10^−3^, 0), (0.26, 1 × 10^−3^, 0.11), (0.26, 1 × 10^−3^, 1), (0.26, 1 × 10^−3^, 2) and (0.26, 1 × 10^−3^, 3) (Sharma et al., 2023). Experimental validation of PID gains is provided elsewhere (Sharma et al., 2023).

## 3 Results

### 3.1 Design and verification of the controller design inputs and outputs

The goal of treating solid tumors, such as GBM, with MHT is to achieve the minimum effective thermal dose (time at temperature) in a maximum of the tumor volume, while minimizing off-target heating. It is often the case in many clinical scenarios that hyperthermia treatments must be completed within about 30 min. This limit on treatment time, combined with a requirement to achieve hyperthermic temperatures within the tumor that must be maintained for the duration of treatment, places significant performance constraints on both device and operators. Though not often used in cancer therapy devices, automated temperature controllers are an established technology designed to achieve demanding criteria.

The US Food and Drug Administration (FDA) regulates design and development of devices intended for treating cancer in human and veterinary settings. For the design and development of the automated controller described here, we implemented recommendations provided in the FDA Device Control Guidance for Medical Device Manufacturers (www.fda.gov) (FDA.gov, 2022). Here we described results of implementing these in the engineering design of an automated temperature-control MHT device. Full verification and validation in a live canine subject is described elsewhere (Sharma et al., 2023).

#### 3.1.1 Experimental electromagnetic and heat transfer model

For verification testing with gel phantoms, our set point temperature was 25 °C to minimize degradation of the gel phantom. Though this temperature is irrelevant for HT, the objective of these verification trials was to verify PID controller performance to specified criteria. These design constraints included a user-defined set point temperature to be achieved rapidly, with minimum overshoot to prevent hotspots, runaway, and off-target heating; and, the user-defined temperature is to be maintained for a period of time determined by the user.

Figure 1B summarizes the controller transient specifications in the *s*-plane. The blue shaded region depicts the region in *s*-space which satisfies these design requirements simultaneously. Design constraints on controller stability mandate that the poles of the transfer function describing the closed-loop system lie on the left-hand side plane of the *y*-axis (LHP). Figure 1B depicts the simulated temperature distribution in the agarose gel + Cu wire system at 30 min.

Experimental open loop heating and cooling temperature vs. time responses to 30 s AMF pulses of fixed amplitudes (53, 123 Oe at 160 kHz), were obtained within the range used for PID control (53–123 Oe peak) (Figure 2A, solid curves). These experiments were conducted using methods described previously (Attaluri et al., 2013). The experimentally measured temperature difference, Δ*T*, was within 5% of the temperature difference predicted by the model (dashed curves, Figure 2A), when the convective heat transfer coefficient, *h*_*conv*_, was assumed to be 21 W·m^−2^·K. Time constants *τ*_1_ and *τ*_2_ and static gain *g* were estimated to define the transfer function of the agarose gel + Cu wire. In Figure 2B, the time constant, *τ*_1_ was measured experimentally (red arrow) as the delay between the maximum rate of change in temperature and the temperature response at the probe location, when applying an AMF pulse with amplitude 9.8 kA/m peak and frequency 160 kHz. The time constant, *τ*_2_, is the difference between the time taken for the temperature response to reach 63% of total temperature gain *τ*_63_ (in s) and the time constant *τ*_1_. We avoided excessively heating the gel sample for long times to prevent its deterioration, hence these time constants *τ*_63_ and *τ*_2_ and gain were estimated by using experimental responses to 30 s AMF pulses and a simulated saturation response from the experimentally validated model to a step increase in magnetic field amplitude (123 Oe, 160 kHz) (Figure 2C). The closed loop temperature vs. time response of gel + Cu wire system with proportional gain was used only to evaluate the undamped natural frequency of the system, *ω*_*n*_ ~ 0.2 rad/s (Figure 2D). Next, the time constants and the undamped natural frequency were used to estimate *K*_*p*_*, K*_*i*_ and *K*_*d*_ gains using Eqs 14–18.

### 3.2 Experimental verification of controller robustness against sensor noise

**FIGURE 3:**
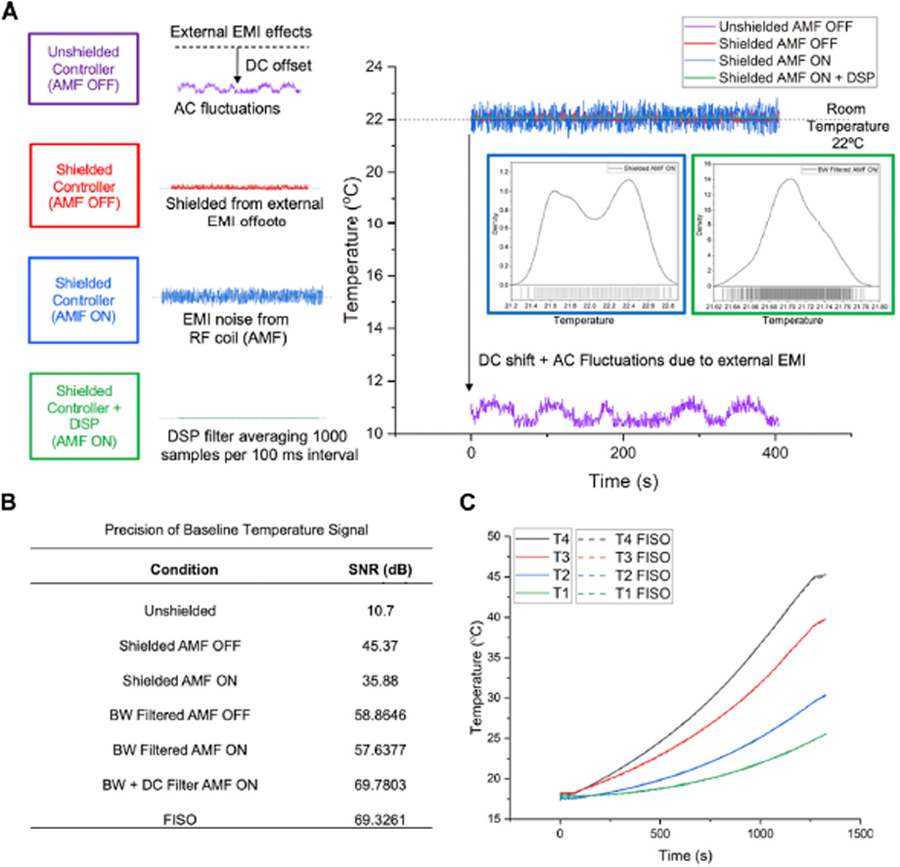
Improvements in precision and accuracy of the digitized probe temperature signal. **(A)** Improvement in signal SNR through mechanical shielding and application of a digital low pass Butterworth (BW) filter. Inset figures in A and Table B show that the low pass filtering generates a Gaussian signal with an improved SNR. All connecting wires were shielded and shielding was electrically grounded to prevent offsets from ground loops. **(B)** Summarized signal precision, measured as SNR in dB, following each signal processing step for the digitized temperature signal vs. FISO reference signal. (C) Accuracy of digitized temperature probe signal in the cRIO controller vs. calibrated temperature signal measured by the FISO SPC-HR reading modules, for all four sensors on the FISO EVO probe, over the temperature range of 20°C–45°C. Solid lines represent measurements taken from the cRIO controller and dashed lines show readouts from FISO SPC-HR reference reading modules.

Results of efforts to shield against RF interference are shown in Figure 3. Mechanical shielding of the controller with a Cu/Ni mesh significantly reduced high frequency noise. Digital signal processing performed in LabVIEW using a digital low-pass Butterworth (BW) filter with cutoff frequency of 100 Hz further improved SNR. Controller code was modified to filter <100 Hz, well below the fastest dynamics of the plant to minimize loss of information. Reduction of the effect of sensor noise through tuning the frequency response of the controller ensured that the controller primarily responded to the user-input set-point and any low-frequency plant disturbances only (Figure 3B). The SNR for the temperature signal measured by the controller was comparable to the FISO reference reader (~69 dB, Figure 3C). The accuracy of the temperature measured by the controller was within 0.3°C of the temperature measured by the FISO reader (Figure 3C dashed lines).

### 3.3 Computational verification with sensitivity analysis and uncertainty propagation

**FIGURE 4:**
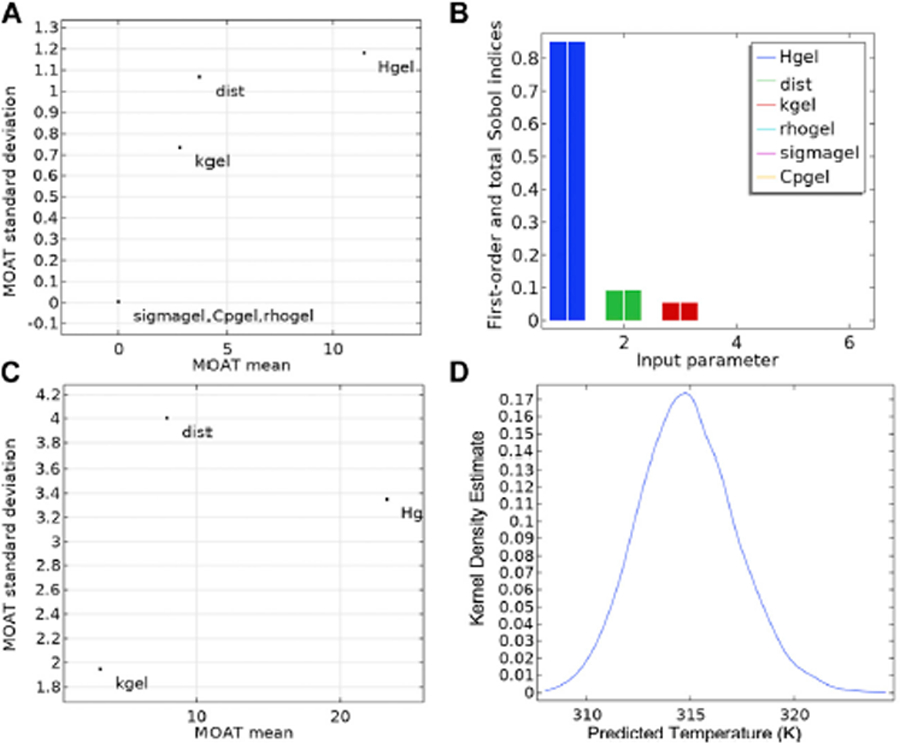
Sensitivity analysis and uncertainty propagation. **(A)** The Morris one-at-a-time (MOAT) method was applied to each input parameter infiuencing the probe temperature at steady state (*t* = 30 min), in the gel + Cu wire experimental setup shown in Figure 1. **(B)** Sensitivity analysis using the Sobol and correlation methods. The first order Sobol index is depicted as the left bar for each parameter in the histogram plot. The total Sobol index, right bar for each parameter in the histogram plot, shows the variance of the probe temperature attributed to the variance of each input parameters and its interaction with the other input parameters. **(C)** The MOAT analysis with higher uncertainty (± 10%) in inputs AMF amplitude (*H*_*gel*_) and probe distance from Cu surface (*dist*). **(D)** Kernel density estimation of the probability distribution function (PDF) of the probe temperature at 1.3 mm from Cu heat source, measured after 10 min of heating. Uncertainties in AMF amplitude and probe distance from the high heating Cu source can result in significant variance in the measured temperature at the probe location.

Figure 4A and Table 3 shows the results of MOAT (Balesdent et al., 2016) analysis, a global sensitivity analysis screening method, in a scatter plot summarizing the relative weights of each input based on their relative effects on the temperature at the sensor location. A high value of the MOAT mean (e.g., *H*_*gel*_) implied that the parameter significantly influenced the temperature. A high value of the MOAT standard deviation implied that the parameter was influential and that it was either strongly interacting with other parameters or that it had a non-linear influence, or both (e.g., *H*_*gel*_ and *dist*). The temperature at the probe location is a non-linear function of the AMF amplitude and probe distance from the heat source, so a strong dependence of temperature on *H*_*gel*_ and *dist* was expected and observed. The thermal conductivity of the gel also influences the temperature at the probe location, while the specific heat, density, and electrical conductivity have much less relative influence on the probe temperature. The MOAT plot is replotted in Figure 4C with higher uncertainty (± 10%) in input AMF amplitude (*H*_*gel*_) and probe distance from Cu surface (*dist*), reflecting experimental uncertainties in AMF amplitude and probe distance.

Figure 4B shows the fractional contribution of each input parameter [Sobol Index (Balesdent et al., 2016)] to the variance of the temperature at the probe location. Sensitivity analysis enabled identification of the set of parameters that most strongly influenced model output. Sobol sensitivity analysis is a global sensitivity analysis method that analyzes and varies all the parameters across their parameter spaces to decompose the model output variance to the relative contributions of individual parameters and their interactions. Sensitivity indices are computed using Monte Carlo methods and this method is excellent for non-linear mathematical models (Sobol, 2001; Zhang, X., et al., 2015).

In our model, the first order Sobol index shows the variance of the probe temperature attributed to each input parameter (Table 4, Table 1) and is depicted as the left bar for each parameter in the histogram plot. Consistent with the MOAT analysis, applied AMF amplitude (*H*_*gel*_), probe distance from heat source (*dist*) and thermal conductivity (*k*_*gel*_) were the major contributors to the variance of the probe temperature, with the strongest influence coming from AMF amplitude (85%). The total Sobol index, right bar for each parameter in the histogram plot, shows the variance of the probe temperature attributed to the variance of each input parameter and its interaction with the other input parameters. The difference between the right and left bars for each parameter is a measure of the contribution of the interaction of that parameter with other input parameters, to the total variance of the probe temperature. Contribution to variance of the probe temperature from interactions between parameters was negligible. Sensitivity of temperature at the probe location was greatest with AMF amplitude, confirming our selection of AMF amplitude as the key control variable.

Figure 4D shows the Kernel density estimation of the probability distribution function (PDF) of the probe temperature at 1.3 mm from Cu heat source, measured at steady state (t = 30 min). As expected, the PDF exhibits a normal distribution from the uncertainties in input parameters, with a mean temperature change (Δ*T*) of 22.87°C, and standard deviation of 2.32°C. We recognize other potential sources of uncertainty include unexpected power supply perturbations, temperature probe motion during measurement, and temperature-dependent non-linear changes to the physical properties of the sample. A complete demonstration of the controller operation is provided elsewhere (Sharma et al., 2023).

### 3.4 Experimental verification of safety controls

**FIGURE 5:**
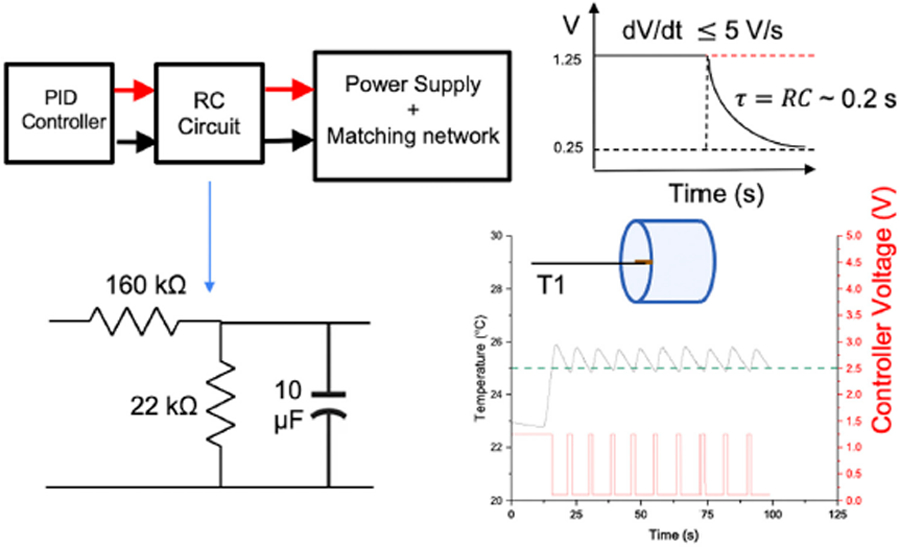
Safety and reliability of the temperature controller. Schematic of RC circuit designed to smooth sharp transitions in voltage. Sharp transitions can induce undesirable power system harmonics that can be damaging to the electronics, or cause power faults. An upper limit to the acceptable rate of change of voltage, dV/dt, was identified as 5 V/s. An RC circuit with a time constant of 0.2 s was sufficient to maintain dV/dt < 5 V/s, for specified upper and lower limits of power supply used for PID control.

To minimize power faults generated by rapid changes in the control signal (*dV/dt* > 5V/s) and to reduce generation of power system harmonics, a RC circuit with a time constant of 0.2 s (Figure 5) was designed and coupled between the controller output and the analog input terminal of the 120 kW AMF. The PID function was programmed as a lower priority than the safety condition, and therefore, is always over-ridden by the safety sensor condition (AMF ON if *T* < *T*_*threshold*_; AMF OFF if, *T* > *T*_*threshold*_). Figure 5 (bottom right) shows this controller action when the safety threshold temperature was set to 25°C. The controller switched off power when the temperature exceeded 25°C (green dotted line) and resumed power when the temperature dropped below the safety threshold. The repetitive action demonstrated the robustness of the safety feature while the RC circuit prevented power supply faults. In this case, the PID controller was untuned to demonstrate this safety action only. More detailed validation of tuned PID temperature control and safety control in a live subject were demonstrated in Sharma et al., 2023.

## 4 Discussion of controller relevance to thermal therapy

The goal of verification experiments described here, and validation experiments described elsewhere (Sharma et al., 2023) was to demonstrate temperature control using the PID controller. For this purpose, any magnetic or metallic material suffices asa heat generator as long as the power deposited with AMF exposure can be calculated or known. NIST traceable Cu wire was chosen as it has defined electrical and thermal properties, and the amount of heat deposited by induction heating can be accurately predicted for a known mass of material and specified AMF conditions (Attaluri, A. et al., 2013). This is impossible for MNPs as no SRM is available for these materials. By way of example for comparison, we demonstrate a calculation based on MNPs in the SM. Experimental demonstration of heating agarose gels with MNPs is also provided in SM (Supplementary Figure S3) showing the heat generated by the Cu wire is relevant for heating with MNPs.

While many studies probe transient characteristics experimentally following controller design (Korganbayev et al., 2020, 2021; Ahmed et al., 2022), in this design we sought to specify target ranges for the transient temperature vs. time characteristics of the controller as design inputs. This was done to exercise greater control of temperature, with a view toward eventual control of the delivered thermal dose, defined using the isoeffect metric CEM43 (Dewey, 2009; Rhoon, 2016), within a defined treatment time (see Supplementary Material S1). The transient characteristics included rise time (*t*_*r*_), settling time (*t*_*ss*_) and overshoot (*M*_*p*_). A short rise time to a hyperthermic set point temperature is desired to optimize time at target temperature in order to achieve the planned thermal dose, i.e., target CEM43. However, power supply faults, causing shutdown, occur if rise time is too short, because high-frequency oscillations of controller voltage ($\omega_{n}\propto \frac{1}{t_{r}}$) are generated by voltage gradients (*dV/dt* > *5 V/s*). We selected a rise time <1 min initially, subject to revision following experimental testing in gel samples with Cu wire heat source.

For many temperature control systems, a slightly underdamped condition (0.7 < *ζ* < 1.0) manifests a temperature vs. time response with an initial overshoot followed by an exponential decay of oscillations (*e*^−*σt*^ · cos *ω*_*d*_*t*). Such a response, coupled with a fast rise time, can maximize CEM43, provided the initial overshoot is not excessive and decays quickly to the target temperature (41°C–46°C). For example, an initial 5% maximum overshoot at 45°C (47.25°C) followed by damping to a maximum of 46°C in 1 min, an acceptable dynamic response results in *σ* = 0.013 s^−1^. A larger *σ* produces faster decay and shorter settling time. Temperature controllers typically display initial overshoot temperatures ranging from 1.8°C–4°C or higher (Korganbayev et al., 2020, 2021). Steady state errors ranging from 0.5°C to 5°C have been reported (Korganbayev et al., 2020; Ahmed et al., 2022). Here, we targeted a maximum initial overshoot <5% (<2.25°C), steady-state error <1% (<0.5°C), and settling time, measured as time to reach target temperature ± 0.5°C. While lower values (<1%) of overshoot and settling time (<1 min) may be desirable and achievable using a single heat source, these are challenging to achieve without causing controller voltage oscillations, especially in cases where multiple heat sources are used (Kandala et al., 2018). Such continuous oscillations can generate damaging power system harmonics.

While the temperature control design parameters were successfully verified here, validation of its performance against intended “end use” criteria was established in a separate series of trials (Sharma et al., 2023). These are briefly summarized here. *In vitro* closed loop experiments to validate performance of controller temperature response were conducted three times (N = 3, at set point 25°C) with gel phantoms. For *ex vivo* validation of hyperthermic temperature control using sections of bovine liver, three experiments were conducted for a model of single heat source (N = 3), and distributed heat sources (N = 3). Within each scenario (single or distributed heat source), each of the three experiments tested the controller performance to achieve different set point temperatures, *T*_*ref*_ = 44°C, 44.5°C and 45°C, respectively. For *in vivo* validation, a total of three short duration AMF pulse tests and three longer temperature-control tests were performed in a live canine research subject. Though CEM43 was not a control parameter, it was evaluated at the end of each validation trial summarized above, by using the entire temperature vs. time data from that trial to retrospectively evaluate the potential for therapeutic benefit of the controlled heating against established clinical benchmark. We might consider a potential benefit by including a CEM43 weighting function in the algorithm to ensure HT remains within a user specified CEM43 while staying within specified temperature limits to ensure safety.

Note that modeling and predicting PID gains works well for temperatures for which the temperature-dependent properties of tissue and blood perfusion are documented and reversible, enabling their inclusion in bioheat transfer modelling (Kandala et al., 2021; Uzuka, T. et al., 2009). On the other hand, modeling effects of irreversible changes that occur with other thermal therapies such as tissue ablation is more challenging because such changes may also be difficult to reproduce. In such cases, PID gains predicted by the model would require separate validation and *in situ* tuning using an adaptive controller that compensates for non-linear and irreversible changes by automatically retuning PID gains or applying a dynamic weighting function to the feedback signal.

## 5 Conclusion

We designed, developed, and evaluated a PID controller device for MHT with an intention to enable future treatment of canine GBM. Careful consideration of safety and performance criteria during device design and development enabled us to minimize operational risk. Specifically, our process placed emphasis on the design inputs, design outputs, and verification process as recommended within the FDA’s Design Control Guidance waterfall method to facilitate device evaluation. Our results demonstrated in gel phantom experiments that the device can automatically adjust the AMF amplitude to maintain the temperature within the target range despite significant perturbations. However, for complex clinical scenarios PID gains may require dynamic adjustment by the operator based on an initial pulse test. Future efforts should explore advanced control strategies such as model predictive control (MPC) for multi-input multi-output (MIMO) systems. Clinical translation of such control systems can improve patient safety and quality assurance.

## Supplementary Material

Published Supplementary Materials

## Figures and Tables

**TABLE 1:** Material properties used in FEA simulations for PID temperature feedback control. Data sourced from Sharma et al. (2023).

Parameters	Mean	Uncertainty
Magnetic Field Amplitude (peak), H_gel_	123 Oe (9.78 kA/m)	±5%
Gel density (rhogel), *ρ*_*gel*_	960 kg/m^3^	±1%
Specific Heat, *C*_*pgel*_	3900 J/(kg.K)	±1%
Thermal Conductivity, *k*_*gel*_	0.566 W/m.K	±1%
Probe distance, *dist*	1.3 mm	±5%
Electrical Conductivity (sigmagel), *σ*_*gel*_	2.1 (S/m)	±5%
Convective heat transfer coefficient (*h*_*conv*_)	21 (W/m^2^.K)	±5%

**TABLE 2:** Parameter list used in simulation of PID gains. Data sourced from Sharma et al. (2023).

Parameter	Value
*τ*_1_	2.96 s
*τ*_63_	252.0 s
*τ*_2_	249.04 s
g	95.83 K/V (23/0.24)
*ω*_*n*_	0.2 rad/s
*ζ*	1
*τ*_*d*_	2.17 s
*κ*	1 × 10^−3^ s^−1^K^−1^
K_p_	0.26 K^−1^
K_i_	1 × 10^−3^ s^−1^K^−1^
K_d_	0.11 s.K^−1^
u_ctrl_	0.24 V
*T*_∞_	294.25 K

**TABLE 3:** Mesh sensitivity analysis performed on agarose gel + Cu wire system. Data sourced from Sharma et al. (2023).

No. of mesh elements	Minimum element size (m)	T_probe_ (^°^C)
53,629	0.0012	50.1
119,110	6.5 × 10^−4^	46.1
181,133	4.5 × 10^−4^	44.6
204,595	4 × 10^−4^	44.2
229,003	3 × 10^−4^	43.8
242,788	2 × 10^−4^	43.7

**TABLE 4:** Cu wire dimensions used for experimental validation of gel + Cu wire system (Sharma et al., 2023).

Materials	Radius (mm)	Length (mm)	Weight (g)
Cu wire	1	4.52	0.104

## Data Availability

The original contributions presented in the study are included in the article/Supplementary Material, further inquiries can be directed to the corresponding author.
