# Supplementary material for: Design of a temperature-feedback controlled automated magnetic hyperthermia therapy device
Source: Front Therm Eng. Author manuscript; Available in PMC 2023 Mar 20. (PMC10026551; doi:10.3389/fther.2023.1131262)
Supplement: Published Supplementary Materials [file NIHMS1881634-supplement-Published_Supplementary_Materials.pdf]

Supplementary Material

Design of a temperature-feedback controlled automated magnetic hyperthermia therapy device

1 Supplementary Information

1.1 Sample calculation of heating with magnetic nanoparticles (MNPs) for comparison with Cu wire

For comparison of calculated heating from MNPs generated by and AMF, we describe below calculations using Synomag-D 70® (Micromod Partikeltechnologied, GmbH) that were used in validation trials (Sharma, et al. 2023). The mass of MNPs required to deposit an equivalent of amount of power as the Cu wire, assuming SLP of Synomag MNPs at the same AMF amplitude (9.78 kA/m and 160 kHz) to be 250 W/g Fe (SI Fig S4) is:

$$P_{SAR}\left(\frac{W}{ml}\right) = SLP\left(\frac{W}{g\ Fe}\right) \times c_{Fe}\left(\frac{g\ Fe}{ml}\right) \quad \text{Eq.1}$$

$$\rho C_p \frac{dT}{dt} = 250 \times c_{Fe}$$

$$8900 \times 385 \times 0.2 \times 10^{-6} = 250 \times c_{Fe}$$

$$c_{Fe} = 2.74\ \mathrm{mg\ Fe/ml}$$

Thus, a uniform MNP concentration of 2.74 mg Fe/ml in the 1 ml gel sample will result in the same temperature rise at the temperature probe as the concentrated Cu heat source. However, if we assume that the MNPs occupy the same volume as the Cu heat source (0.014 ml), then the concentration of iron required, $c_{Fe} = \frac{2.74}{0.014} \sim 195\ \mathrm{mg\ Fe/ml}$. Thus, knowledge of MNP distribution is essential to determine the accurate concentration required for thermal treatments. For in vivo validation studies, we infused MNPs with $c_{Fe} = 80\ \mathrm{mg}\frac{Fe}{ml}$ locally by percutaneous injection (Sharma et. al., 2023).

1.2 Thermal dosimetry and CEM43 calculations from experimental temperature data

The thermal isoeffect dose, Cumulative Equivalent Minutes at 43 °C, or CEM43, can be evaluated by using the entire temperature vs time data from each trial and applying the formula:

$$\mathrm{CEM43} = \sum \Delta t \cdot R^{43-T} \quad \text{Eq.2}$$

where Δt is the time interval for each measurement in s, T is the measure temperature in °C, and R is an empirical, species-specific dimensionless constant (Franckena, M. et al., 2009). Our control algorithm did not use CEM43 as a control parameter. Instead, feedback was based on the difference between the value of the measured temperature at the sensor location and the user-specified set point hyperthermic temperature because safety (e.g. pain or burns) depend on temperature.

1.3 Pulse-width modulation (PWM) controller

The PID controller was the main controlling entity of the device algorithm described here. A simple PWM controller was also integrated as back up to the PID controller (in case PID became unstable, i.e., plant mismatch). The PWM controller used the principle that whenever the temperature sensed by the probes exceeded the set point temperature, power supply was shut off discontinuing heating. As the temperature fell below the set point, the PWM controller then enabled power to resume heating. As the operating range of the PWM controller was set to occur within two voltage limits, i.e., user defined high and low voltages, the output of the PWM controller was a pulsed waveform.

2 Supplementary Figures

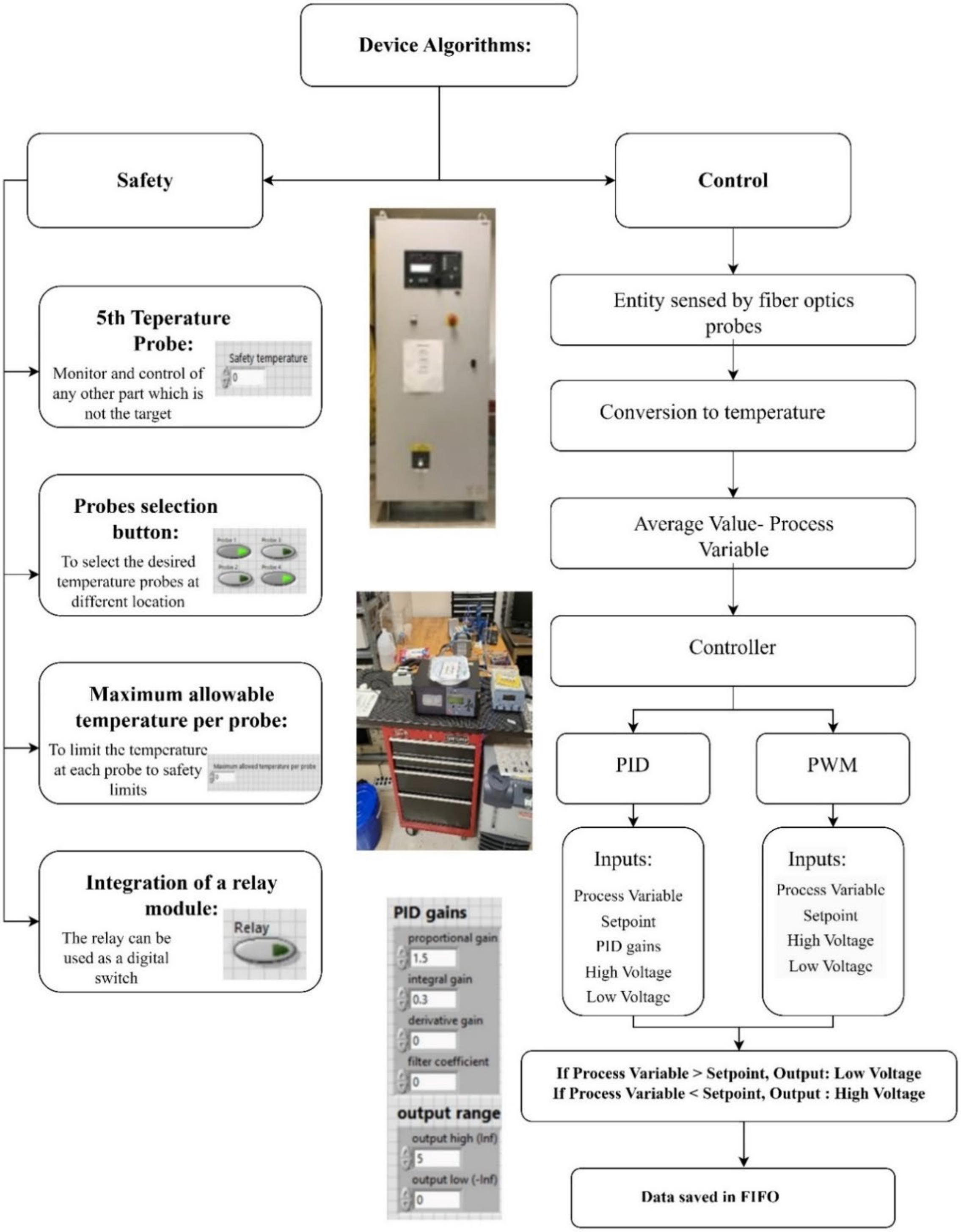

Supplementary Figure 1. MHT device algorithm with safety temperature thresholds and target temperature control. In the present work, we describe design, build, and design verification testing of a PID controller. Pulse-width modulation (PWM) was integrated as backup to the PID controller.

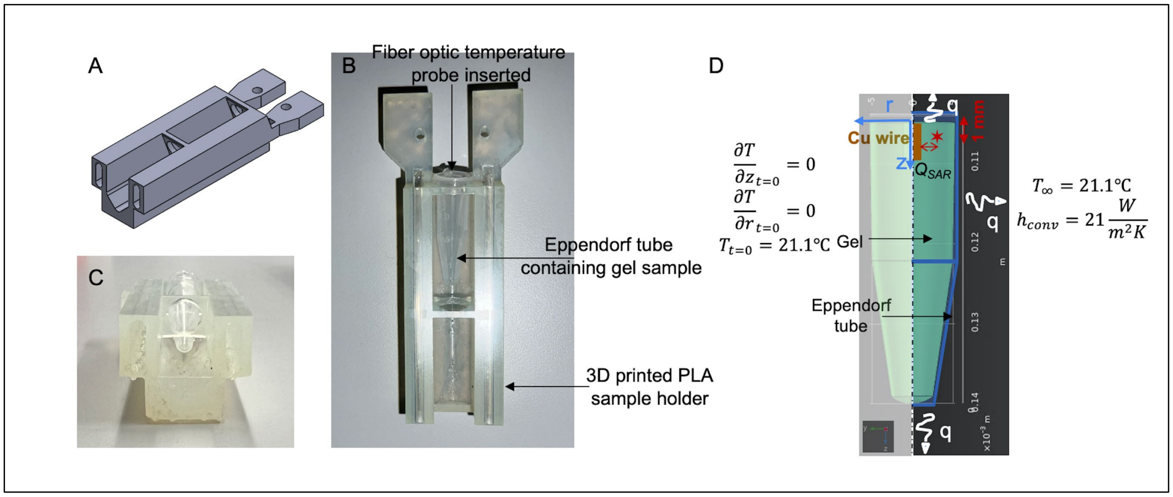

Supplementary Figure 2. (A) Schematic showing the geometry of the 3D printed PLA sample holder used for calorimetric *in vitro* studies. (B, C) Photographs of PLA sample holder and Eppendorf tube containing gel sample positioned centrally in the sample holder. (D) Schematic of agarose gel + Cu wire sample with boundary and initial conditions. The red star marks the position of the fiberoptic temperature sensor placed 1.3 mm from the Cu wire surface and a 1 mm depth. "q" shows the convective heat loss to the environment and Q_{SAR} is the power density deposited by the Cu wire heat source.

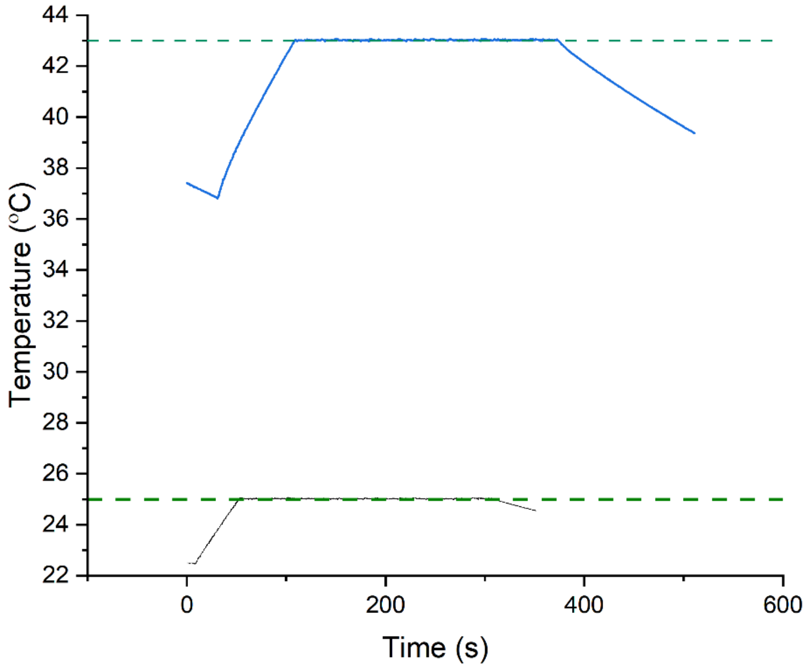

Supplementary Figure 3. Closed-loop temperature vs time response in agarose gels containing magnetic nanoparticles uniformly distributed (cFe~2.5 mg Fe/ml, Synomag, Micromod Partikeltechnologie, GmbH) for two separate setpoints (25°C and 43°C, dashed green lines) using PID-based temperature feedback control. 25°C response was also shown previously in Jangam *et al.* (2022).

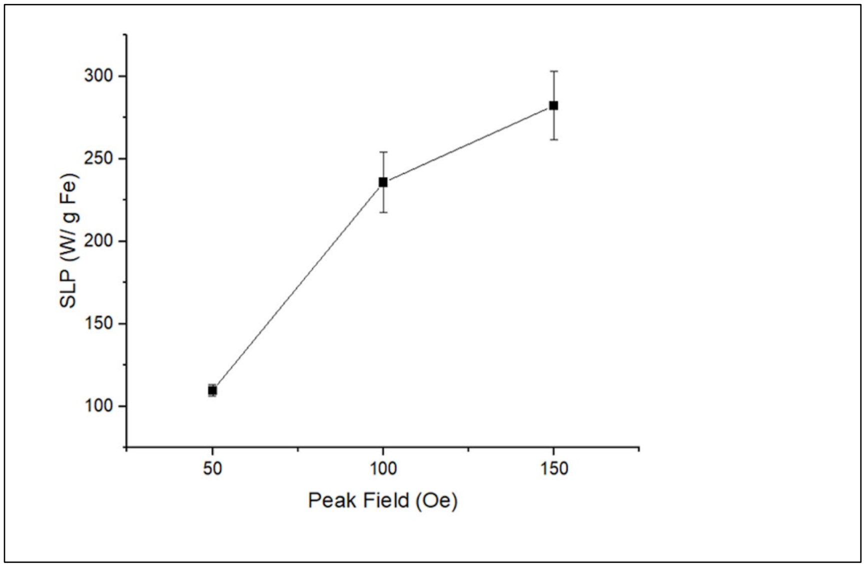

Supplementary Figure 4. Non-linear AMF amplitude-dependent specific loss power (SLP) of Synomag nanoparticles (*cFe of 2.5 mg Fe/ml) over the AMF amplitude range of interest (50-150 Oe peak; and 160 kHz) allows application of AMF amplitude-modulated methods (like PID control) to control tumor temperature and thermal dose, CEM43
